# Supine Percutaneous Nephrolithotripsy in Double-S Position

**DOI:** 10.1155/2018/7193843

**Published:** 2018-03-11

**Authors:** Giuseppe Giusti, Antonello De Lisa

**Affiliations:** Department of Urology, University of Cagliari, Via Is Mirrionis 92, 09121 Cagliari, Italy

## Abstract

**Background:**

At present, the percutaneous nephrolithotripsy (PCNL) is performed both in supine and in prone position. The aim of this paper is to describe an innovative position during PCNL.

**Methods:**

We describe a supine position. The patient's legs are slightly abducted at the hips. The thorax is laterally tilted (inclination 30°–35°) and kept in the right position by one or two gel pads placed between the scapula and the vertebrae. External genitalia can be accessed at any time, so that it is always possible to use flexible instruments in the upper urinary tract. We used this position for a period of 12 months to treat with PCNL 45 patients with renal lithiasis.

**Results:**

All the procedures were successfully completed without complications, using the position we are describing. The following are some of its benefits: an easier positioning of the patient; a better exposure of the flank for an easier access to the posterior renal calyces of the kidney; a lower risk of pressure injuries compared to positions foreseeing the use of knee crutches; the possibility of combined procedures (ECIRS) through the use of flexible instruments; and a good fluoroscopic visualization of the kidney not overlapped by the vertebrae.

**Conclusions:**

This position is effective, safe, easy, and quick to prepare and allows for combined anterograde/retrograde operations.

## 1. Background

Percutaneous access to the kidney was first used in 1954 when radiologists dared to puncture the pelvis of hydronephrotic kidneys to perform anterograde pyelography [1]. The techniques to place nephrostomy drainages have been developed over the years, as well as the technique to extract stones from the kidney's cavities (percutaneous nephrolithotripsy (PCNL)) [2].

The traditional prone position has in fact been developed by radiologists, who used to puncture the pelvis directly, and not through the renal parenchyma. In 1987-88, Valdivia et al. [3] reported a safe percutaneous access to the kidney with a supine patient, and 10 years later they reported the *in vivo* experience with 557 patients [4]. The supine position became more popular after 2007 when the Galdakao modification was reported in an international publication [5].

The advantages of percutaneous nephrolithotripsy (PCNL) performed in the supine position are both of an anesthesiological and urological kind. The surgeon does not experience the cardiovascular [6], ventilatory, neuroendocrine, and pharmacokinetic problems of the prone position in the supine ones that grant better access to the airways and the cardiovascular system. Urological advantages include an easier puncture of the kidney which lies closer to the skin; a demonstrated decreased risk of colon injury [7]; a better descending drainage and retrieval of stone fragments, facilitated by the downward position of the Amplatz sheath; and a lower intrarenal pressure leading to smaller pyelovenous back flow and postoperative infectious risk [8].

Authors have suggested many different versions of the supine and prone positions to optimize the technique: the lateral [9], laterally flexed [10], lateral with abducted legs [11], prone split leg [12], prone-reverse lithotomy [13], supine crossed-leg [14], and supine with legs flexed in supports [3] are only some of them.

In our department, we perform an average of 79 percutaneous nephrolithotripsy per year. The need for an easy and quick-to-prepare position, which could at the same time allow for a safe and effective execution of the procedure, led us to develop the supine double-S (Slightly tilted thorax-Slightly split legs) position, which will be described in the following lines.

## 2. Materials and Methods

This is a preliminary study, carried out by the Department of Urology at the University of Cagliari (Cagliari, Italy) with the purpose to first describe the innovative position we developed.

The objective of the study was explained to all enrolled patients. Informed consents to participate and use personal data and images were signed by all enrolled patients.

For every case study, we recorded the patient's BMI, the dimension and the position of the stones, the operating time, and the complications occurred (using the Clavien-Dindo classification).

### 2.1. Description of the Position

The patient is placed on the operating table with the glutei at the lower end of the bed and the external genitalia exposed for an easy access.

**Figure 1:**
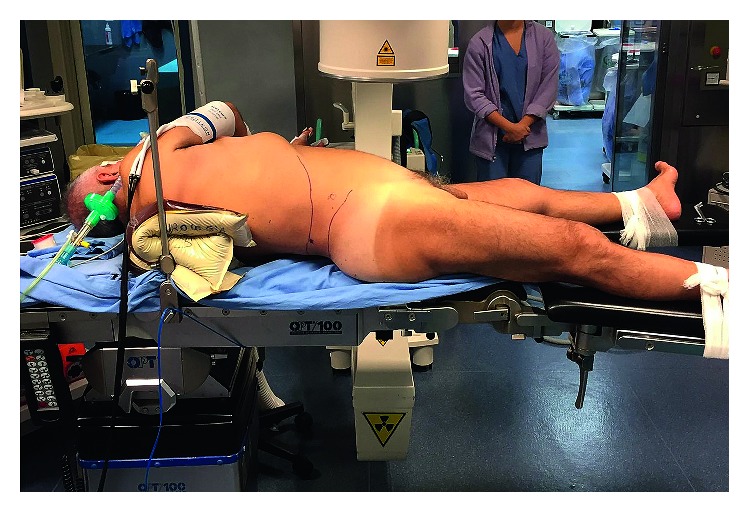
Patient's position on the operating table.

**Figure 2:**
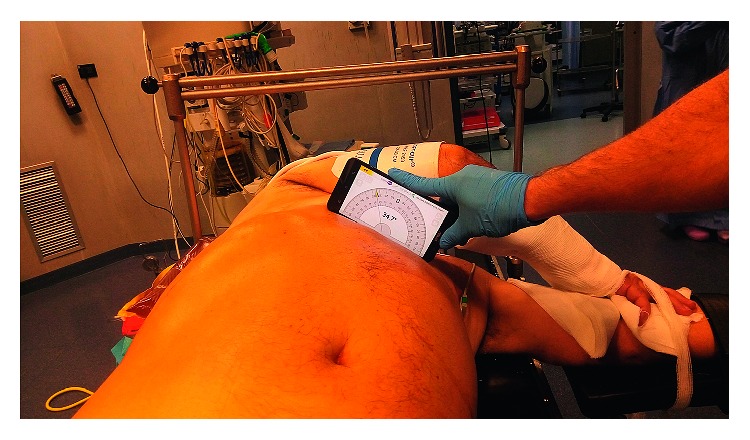
Measurement of the inclination of the patient's thorax through the app Protractor.

It is important not to place the patient too close to the metallic side of the table, which is radiopaque and may project on the fluoroscopic field, obscuring the images. The patient's legs are secured independently and slightly abducted at the hips without being flexed (Figure 1). This position of the legs allows for retrograde access to the bladder and the upper urinary tract using flexible instruments. The ipsilateral arm is placed on the thorax and fixed to the contralateral one, by means of a wrist bandage (Figure 2). The contralateral arm is abducted and placed on a support. The thorax is slightly tilted. Its inclination, between 30° and 35°, can be measured by placing a smartphone over the body of the sternum, using Protractor (ExaMobile S.A., Poland—available on Google Play Store), an application which exploits the phone's accelerometers (Figure 2). The position is secured by one or two gel pads placed between the ipsilateral scapula and the spine. The positioning of the anaesthesia screen at the level of the interscapular line prevents the pads from moving. The position offers a good exposure of the flank to be treated. The retrograde access to the urinary tract is always possible through the use of flexible instruments (with or without the use of the ureteral sheath).

**Figure 3:**
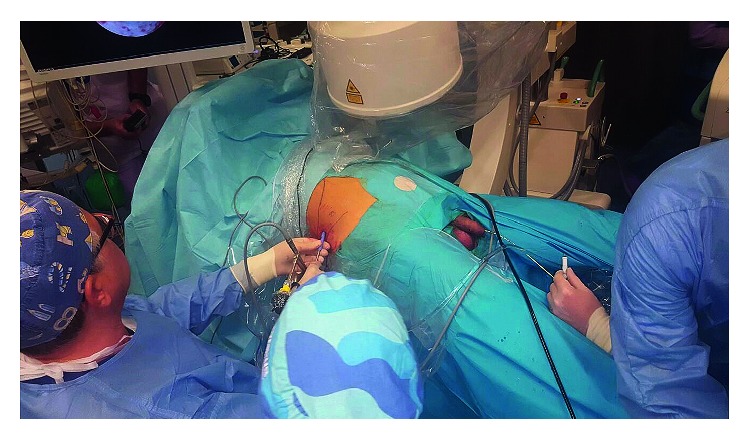
The procedure. The assistant has access to the upper urinary tract by standing between the patient's legs.

The renal puncture and the remaining phases of the surgery do not differ from the traditional procedure in the Valdivia position (Figure 3).

Between March 1, 2016, and March 1, 2017, the double-S position was used to treat 45 patients.

Patients presented an average body mass index (BMI) of 27.4.

In the CT (computed tomography) study, the mean stone size was 2.2 cm. The stones were positioned in the renal pelvis (48.8%), in the inferior calyx (22.2%), and in the medium calyx (8.8%). No staghorn stones were documented. In 9 cases, we treated multicalyceal stones (20%). In these 9 cases, we preferred not to perform a secondary access but a combined anterograde-retrograde procedure using a flexible ureteroscope. When possible, the stone was relocated in a place where lithotripsy could be performed through the percutaneous access; when this maneuver was not feasible, the stone was shattered using laser energy.

Patients were treated with an antibiotic prophylaxis before any procedure.

The effectiveness of all the operations was evaluated on the basis of the stone-free status after one month from the procedure, ascertained with CT abdomen examination without medical contrast medium (stone-free status < 3 mm).

## 3. Results

All the procedures performed in the described position were successfully completed. The mean operating time was 43.5 minutes.

At the end of each operation, a flexible pyelocalyceal-scopy and an anterograde pyelogram were performed, and in all cases the patients resulted 100% stone-free. With a CT abdomen without medical contrast medium after one month, we had the confirmation of the stone-free status for all operations.

There were neither high (III–V) nor low level (I-II) complications according to Clavien-Dindo.

## 4. Discussion

Besides the well-known anesthesiological advantages of the supine position, the double-S position presents other benefits. It indeed combines all the advantages of the several variations of the supine position already described in the scientific literature [15–17]:

- A smaller number of nurses in the operating room, who have an easier position to prepare and lighter loads to shift.
- A better exposure of the flank and an increased distance between the last rib and the iliac crest (as reported by Desoky), providing a wider space for puncture, dilatation, and maneuverability of the nephroscope. This is feasible thanks to the fact that a single support is placed under the shoulder, and not under the lumbar region as in the Valdivia position (Figure 1).
- A lower risk of pressure injuries to vascular and nervous structures compared to positions foreseeing the use of knee crutches (e.g., Galdakao position).
- The absence of flank support, which prevents the cephalad sliding of the kidney, making upper-pole puncture more feasible.
- A lower degree of thorax rotation, allowing for a better fluoroscopic view of the kidney, which is not overlapped by the vertebrae, as it may happen in semi-supine (more tilted) positions.
- The possibility to always have a retrograde access to the high urinary tract.

Someone could state that the lack of space between the legs, as they are only slightly abducted, could represent an issue for the assistant carrying out the retrograde intrarenal procedure. We, however, did not experience such a drawback. For every retrograde procedure, we chose the use of a ureteral sheath that allowed for an easy access and the possibility, when necessary, of multiple entries and exits. Sometimes, with female patients, we had to ask for the assistance of the scrub nurse to find the urethral meatus. Even in those cases, however, after the positioning of the sheath, the access was always easy.

A similar supine position has been reported by Desoky et al. and published in the *Arab Journal of Urology* in 2012 [18]. Desoky's position differs, however, from the double-S for the positioning of the patient's legs—slightly split in ours, and crossed in the flank-free position he described—and does not allow for the use of retrograde access in the upper urinary tract, which is instead always possible in ours.

Another similar position is the Barts “flank-free” modified supine one [19], which differs from the double-S for the necessary use of stirrups and the placement of a second support under the gluteus. These supplementary requirements take a long time. Our position is quicker to prepare and not as dangerous as those entailing a wider abduction of the legs, especially in patients with hip joint and femur problems.

## 5. Conclusions

The supine double-S position we describe turned out to be efficient, safe, easy, and quick to prepare. It allows for an easy access to the upper urinary tract and therefore for the performance of combined anterograde/retrograde operations. In our clinical practice, it has become the standard position for PCNL procedures in a supine position. In the age of flexible endoscopy, we believe that this position could be a new valid option and we recommend it both to those with a good experience in percutaneous kidney operations as well as to those who are approaching this technique.

## Data Availability

The datasets used and/or analyzed during the current study are available from the corresponding author on reasonable request.

## Abbreviations

PCNL: Percutaneous nephrolithotripsy
ECIRS: Endoscopic combined intrarenal surgery.
